# Effect of glucagon-like peptide-1 receptor agonists on major adverse cardiovascular events in adults with type 2 diabetes and established atherosclerotic cardiovascular disease: systematic review and meta-analysis of randomised trials

**DOI:** 10.1097/XCE.0000000000000360

**Published:** 2026-06-26

**Authors:** Alejandro Montenegro-Avila, Abelardo Montenegro-Cantillo, Jheyson Fuentes, Jennifer Cifuentes, German Camilo Giraldo-Gonzalez

**Affiliations:** a Universidad de Manizales; b Todo por el Corazon, Cardiology Unit, Manizales; c Clínica Internacional de Alta Tecnología en Cáncer (Clinaltec), Ibagué, Colombia

**Keywords:** cardiovascular outcomes, complications macrovascular, glucagon-like peptide-1 receptor agonists, major adverse cardiovascular events, meta-analysis, systematic review, type 2 diabetes

## Abstract

Cardiovascular outcome trials suggest that glucagon-like peptide-1 receptor agonists (GLP-1RAs) reduce major adverse cardiovascular events in individuals with type 2 diabetes (T2DM), but the magnitude of benefit, including recent evidence from the landmark SOUL trial and oral formulations, remains uncertain. We performed a Preferred Reporting Items for Systematic Reviews and Meta-Analyses 2020 (International Prospective Register of Systematic Reviews ID: CRD420251034652), searching PubMed, Embase, Cochrane CENTRAL, Scopus and ClinicalTrials.gov on 6 May 2025 for randomised controlled trials comparing GLP-1RAs with placebo or usual care in adults with T2DM and established atherosclerotic cardiovascular disease. Seven trials, including 56 191 participants (mean follow-up: 3.5 years), were analysed. GLP-1RAs reduced major adverse cardiovascular events by 11% [hazard ratio: 0.89, 95% confidence interval (CI): 0.83–0.96], all-cause mortality by 11% (hazard ratio: 0.89, 95% CI: 0.82–0.97) and hospitalisation for heart failure by 7% (hazard ratio: 0.93, 95% CI: 0.89–0.98), all with high-certainty evidence. Overall, GLP-1RAs – available in subcutaneous and oral formulations – provide clinically meaningful cardiovascular benefits in high-risk adults with T2DM.

## Introduction

The rising prevalence of type 2 diabetes (T2DM) poses a major public health challenge, particularly given its close association with atherosclerotic cardiovascular disease (ASCVD). Approximately 80% of individuals with diabetes die from cardiovascular causes [1], underscoring the urgency of effective risk-reduction strategies. Glucagon-like peptide-1 receptor agonists (GLP-1RAs) have emerged as a promising class in this context: recent cardiovascular outcome trials suggest they reduce major adverse cardiovascular events (MACE) by approximately 12–14% in patients with T2DM and established ASCVD [2], while also enhancing glycaemic control, promoting weight loss and providing direct cardioprotective effects [3,4–6].

Despite this evidence, important questions remain. The extent to which cardiovascular benefit is shared across structurally distinct agents – human-sequence analogues versus exendin-4 derivatives – and across different routes of administration has not been fully characterised. The oral formulation of semaglutide offers an attractive option for patients who prefer to avoid injectable therapy, and cardiovascular outcome data for this route are now available with the recent SOUL trial. This systematic review and meta-analysis, therefore, aims to assess the efficacy of GLP-1RAs on cardiovascular outcomes in adults with T2DM and established ASCVD, incorporating the most recent evidence to inform optimal therapeutic decision-making in this high-risk population.

Unlike prior meta-analyses, the present review incorporates the recently published SOUL trial, thereby updating the cardiovascular evidence base for GLP-1RAs with the first large, event-driven trial demonstrating macrovascular efficacy of an oral GLP-1RA formulation. This is clinically relevant because GLP-1RAs differ not only in molecular origin – human-sequence versus exendin-based compounds – but also in pharmacokinetic profile and route of administration, and the available evidence does not support assuming a uniform class effect [7].

## Methods

### Protocol and registration

The protocol for this review was prospectively registered in the International Prospective Register of Systematic Reviews (ID: CRD420251034652) on 17 April 2025 (available at https://www.crd.york.ac.uk/PROSPERO/view/CRD420251034652). The protocol prespecified the research question, eligibility criteria, search strategy, risk-of-bias assessment and analytical plan. No deviations from the published protocol occurred.

### Reporting guideline

The review and meta-analysis is reported in accordance with the Preferred Reporting Items for Systematic Reviews and Meta-Analyses 2020 statement [8] , and the completed checklist is provided in Supplementary Appendix 1, Supplemental digital content 1, https://links.lww.com/CAEN/A80.

Eligibility criteria were:

- randomised controlled trials (RCTs) enrolling adults (≥ 18 years) with T2DM and documented ASCVD and
- intervention with any GLP-1RAs (e.g. liraglutide, semaglutide, dulaglutide and exenatide) at any dose or frequency.

Full‑text screening was restricted to English and Spanish articles because more than 95% of cardiovascular outcomes trials are published in these languages. A pilot screening of titles and abstracts in other languages identified no potentially eligible studies; hence, this limitation was deemed unlikely to introduce meaningful exclusion bias.

### Outcomes

The primary outcome was MACE, defined as the composite of cardiovascular death, nonfatal myocardial infarction (MI) and non-fatal stroke. Secondary outcomes included all-cause mortality, individual cardiovascular events (MI and stroke) and hospital admission for heart failure.

### Information sources and search strategy

Systematic research was conducted in PubMed/MEDLINE, Embase, Cochrane CENTRAL, Scopus and ClinicalTrials.gov. No date limits were applied; the final search was run on 6 May 2025. Search strategies combined controlled vocabulary (e.g. medical subject headings and Emtree) and free-text terms for ‘GLP-1 receptor agonists’, ‘type 2 diabetes’ and ‘cardiovascular events’. The complete strategies are provided in Supplementary Appendix 1, Supplemental digital content 1, https://links.lww.com/CAEN/A80. Additional records were identified by snowballing reference lists of eligible studies and relevant reviews.

### Study selection

All retrieved records were imported into EndNote for de-duplication and then screened in Rayyan. Two reviewers independently examined titles and abstracts, followed by full-text assessment for eligibility. Discrepancies were resolved through discussion or, when necessary, consultation with a third reviewer.

### Data extraction

Data were extracted with a standardised Excel form and included study characteristics (author, year, country and design), participant demographics (age, sex and baseline cardiovascular status), intervention and comparator details, outcomes, effect estimates, follow-up duration, funding sources and conflicts of interest.

### Risk of bias

Risk of bias was assessed independently by two reviewers using the Cochrane Risk of Bias 2 (RoB 2) tool [9], which evaluates five domains. Any disagreements were resolved by consensus.

### Data synthesis and statistical analysis

Effect estimates were expressed as hazard ratios with 95% confidence intervals (CIs). For meta-analysis, hazard ratios were log-transformed and pooled using an inverse-variance random-effects model to account for potential clinical heterogeneity. Between-study variance was estimated with restricted maximum likelihood, and pooled estimates were back-transformed to hazard ratios with 95% CIs. Heterogeneity was assessed using *I*^2^ and *τ*^2^, interpreted alongside clinical differences across trials; *I*^2^ values were considered moderate at 30–60%, substantial at 50–90% and considerable above 75%.

Prespecified subgroup analyses were undertaken to explore sources of heterogeneity and to assess whether treatment effects varied by clinically relevant factors. These analyses stratified trials by: GLP-1RA backbone: human-sequence analogues (liraglutide, semaglutide and dulaglutide) versus exendin-4 derivatives (exenatide and lixisenatide), owing to their structural and pharmacodynamic differences. Follow-up duration was less than 3 years versus greater than or equal to 3 years, recognising that a longer exposure may be required to observe cardiovascular benefit. Concomitant cardiovascular therapies were use of statins, angiotensin-converting enzyme inhibitors or angiotensin II receptor blockers, and sodium-glucose co-transporter 2 inhibitors. Publication bias was evaluated by visual inspection of funnel plots.

### Certainty of evidence

The certainty of evidence was assessed using the Grading of Recommendations Assessment, Development and Evaluation (GRADE) framework across five domains: risk of bias, inconsistency, indirectness, imprecision and publication bias. Certainty was rated as high, moderate, low or very low according to the presence and severity of concerns in these domains [10] . Downgrading decisions were mainly driven by imprecision, when CIs crossed or approached the line of no effect, and by inconsistency, when heterogeneity was moderate or clinically unexplained. Risk of bias was not considered serious because all included trials were judged to be at low risk using RoB 2, and indirectness was not considered serious because the populations, interventions, comparators and outcomes were aligned with the review question. Publication bias was assessed qualitatively through funnel-plot inspection, given the small number of included studies. Detailed judgements for each outcome are provided in the Supplementary material, Supplemental digital content 1, https://links.lww.com/CAEN/A80.

## Results

**Fig. 1:**
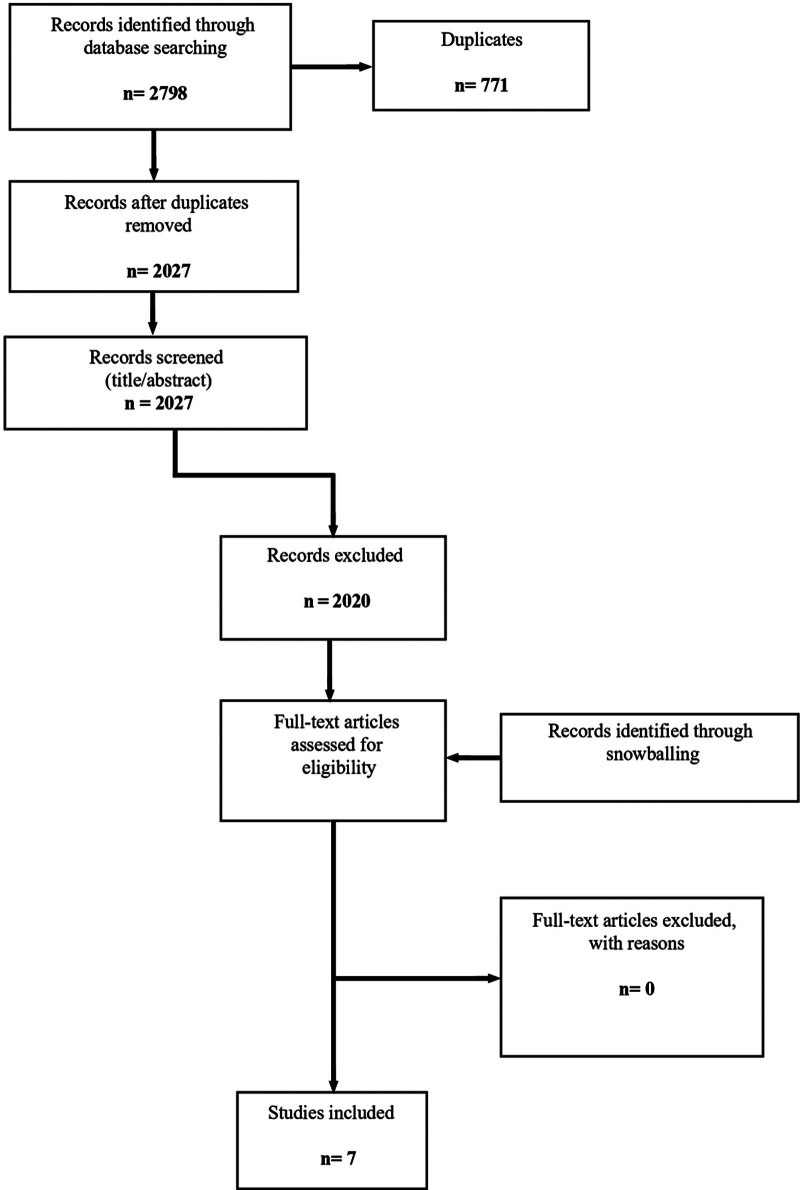
PRISMA 2020 flow diagram of study selection. The diagram details the identification, screening, eligibility and inclusion of RCTs evaluating GLP‑1 receptor agonists in adults with type 2 diabetes and established ASCVD. Reasons for exclusion are listed at each stage. ASCVD, atherosclerotic cardiovascular disease; PRISMA, Preferred Reporting Items for Systematic Reviews and Meta-Analyses; RCTs, randomised controlled trials.

The search yielded 2798 records across PubMed, Embase/ClinicalTrials, Scopus and Cochrane CENTRAL (no date limit; restricted to English and Spanish). After removing 771 duplicates, 2027 records remained for title and abstract screening. Of these, 2020 were excluded for not meeting eligibility criteria. Seven full-text articles were assessed and all fulfilled the inclusion criteria; no additional studies were identified by snowballing. Consequently, seven RCTs were included. The Preferred Reporting Items for Systematic Reviews and Meta-Analyses flow diagram is shown in Fig. 1.

All included trials were multicentre, phase 3 or 4 RCTs evaluating the cardiovascular safety and efficacy of GLP-1RAs in adults with T2DM and established ASCVD. Table 1 summarises key characteristics, including participant demographics, intervention and comparator details, follow-up duration and funding sources.

**Table 1:** Characteristics of randomised controlled trials evaluating glucagon-like peptide-1 receptor agonists in adults with type 2 diabetes and established atherosclerotic cardiovascular disease

Author and year	Gerstein *et al*., 2019 [11]	Holman *et al*., 2017 [12]	Husain *et al*., 2019 [13]	Marso *et al*., 2016 [14]	Marso *et al*., 2016 [19]	McGuire *et al*., 2025 [7]	Pfeffer *et al*., 2015 [15]
Study name	REWIND	EXSCEL	PIONEER 6	LEADER	SUSTAIN-6	SOUL	ELIXA
Study design	Parallel, multicentre, double-blind, placebo‑controlled RCT	Parallel, multicentre, double-blind, placebo‑controlled RCT	Parallel, multicentre, double‑blind, placebo-controlled RCT	Parallel, multicentre, double-blind, placebo‑controlled RCT	Parallel, multicentre, double-blind, placebo‑controlled RCT	Parallel, multicentre, double-blind, placebo‑controlled RCT	Parallel, multicentre, double-blind, placebo‑controlled RCT
Country or región	371 sites in 24 countries (including Canada, Argentina, India, USA, Brazil, Spain, Colombia and others)	687 centres in 35 countries (including the USA, Europe, Latin America and Asia)	21 countries (North America, Europe and Asia)	410 centres in 32 countries	20 countries (Europe, North America, Asia and Oceania)	32 countries	49 countries across the Americas, Europe, Asia, Africa and Oceania
Follow-up duration	Median: 5.4 (IQR: 5.1–5.9) years	Median: 3.2 (IQR: 2.2–4.4) years; up to 7 years	Median: 15.9 months	Median: 3.8 years	Median: 2.1 years	Median: 3.4 years	Median: 2.1 years
Sample size	9901 participants (4949 dulaglutide/4952 placebo)	14 752 participants (7356 exenatide/7396 placebo)	3183 participants (1591 semaglutide/1592 placebo)	9340 participants (4668 liraglutide/4672 placebo)	3297 participants (1648 semaglutide/1649 placebo)	13 206 participants (6603 oral semaglutide/6603 placebo)	6068 participants (3034 lixisenatide/3034 placebo)
Mean age (years)	66.2 (SD: 6.5)	61.8 (SD: 9.8)	66 (SD not specified)	64.3 (SD: 7.2)	64.6 (SD: 7.2)	69.5	60.3 (SD: 9.2)
Female (%)	46.3	38.6	31.6	35	39	34.7	31.4
Inclusion criterion	T2DM ≥ 50 years, HbA1c ≤ 9.5%, BMI ≥ 23 kg/m^2^, with prior CVD or age‑related risk factors	T2DM ≥ 18 years, HbA1c 6.5–10%, with or without established CVD	T2DM ≥ 50 years with established CVD or ≥60 years with cardiovascular risk factors	T2DM ≥ 50 years with established CVD or ≥ 60 years with ≥1 cardiovascular risk factor	T2DM ≥ 50 years with established CVD or ≥60 years with ≥1 cardiovascular risk factor	Adults with T2DM and established CVD, or chronic kidney disease with target‑organ damage (albuminuria, LVH, etc.)	T2DM with a recent acute coronary event (14–180 days before randomisation)
Exclusion criterion	eGFR < 15 ml/min/1.73 m^2^, cancer within the past 5 years, severe hypoglycaemia, life expectancy < 1 year, cardiovascular event < 2 months, scheduled surgery	Recent cancer (<5 years), previous pancreatitis, severe gastrointestinal disease, advanced hepatic or renal insufficiency (eGFR < 30 ml/min/1.73 m^2^), life expectancy < 1 year	Severe hepatic failure, eGFR < 30 ml/min/1.73 m^2^, previous pancreatitis, active cancer, pregnancy	Severe hepatic or renal failure, active cancer, previous pancreatitis, life expectancy < 2 years	eGFR < 30 ml/min/1.73 m^2^, previous pancreatitis, active cancer, severe liver disease	Hepatic insufficiency, previous pancreatitis, active cancer, proliferative diabetic retinopathy	Advanced renal failure, severe liver disease, previous pancreatitis, recent bariatric surgery, limited life-expectancy
Established CVD (%)	31.5	73.1	84.7	81.3	83	99	100 (all participants had experienced a recent acute coronary syndrome)
Intervention	Dulaglutide 1.5 mg subcutaneously weekly + usual care	Exenatide 2 mg subcutaneously weekly + standard care	Oral semaglutide 14 mg once daily + standard care	Liraglutide 1.8 mg SC once daily (titrated from 0.6 mg) + usual care	Semaglutide SC weekly (0.5 mg or 1.0 mg; titrated)	Oral semaglutide 14 mg once daily + standard care	Lixisenatide 20 μg SC daily (titrated from 10 μg)
Comparator	Placebo subcutaneously weekly + usual care	Placebo subcutaneously weekly + standard care	Placebo oral once daily + standard care	Placebo SC once daily + usual care	Placebo SC weekly	Placebo oral once daily	Placebo SC daily
Concomitant medications	Statins 66.3%; ACE inhibitors/ARBs 81%; insulin 24%; sulfonylureas 45.9%; metformin 81.3%	Metformin 77%; sulfonylureas 41%; insulin 46%; statins 75%; ACE inhibitors/ARBs 79%	Metformin 73.2%; sulfonylureas 43.4%; insulin 33.9%; statins 75.2%; ACE inhibitors/ARBs 82.3%	Metformin 76%; insulin 63%; sulfonylureas 49%; statins 72%; ACE inhibitors/ARBs 88%	Metformin 73%; sulfonylureas 43%; insulin 58%; statins 76%; ACE inhibitors/ARBs 87%	Statins 78.1%; ACE inhibitors/ARBs 86.2%; metformin 60.6%; insulin 41.2%; SGLT2 inhibitors 20.4%	Basal insulin 39%; metformin 66%; sulfonylureas 30%; statins 90%; ACE inhibitors/ARBs 88%
Primary outcome (MACE)	HR: 0.88, 95% CI: 0.79–0.99	HR: 0.91, 95% CI: 0.83–1.00	HR: 0.79, 95% CI: 0.57–1.11	HR: 0.87, 95% CI: 0.78–0.97	HR: 0.74, 95% CI: 0.58–0.95	HR: 0.84, 95% CI: 0.74–0.97	HR: 1.02, 95% CI: 0.89–1.17
Secondary outcomes	CV death: HR: 0.91 (0.78–1.06), *P* = 0.21; non-fatal MI: HR: 0.96 (0.79–1.16), *P* = 0.65; non‑fatal stroke: HR: 0.76 (0.61–0.95), *P* = 0.017; all‑cause death: HR: 0.90 (0.80–1.01), *P* = 0.067; HF hospitalisation/urgent visit: HR: 0.93 (0.77–1.12), *P* = 0.46; unstable‑angina hospitalisation: HR: 1.14 (0.84–1.54), *P* = 0.41	All-cause death: HR: 0.86 (0.77–0.97); CV death: HR: 0.88 (0.76–1.02); MI (fatal + non‑fatal): HR: 0.97 (0.85–1.10); fatal MI: HR: 1.29 (0.63–2.66); stroke (fatal + non‑fatal): HR: 0.85 (0.70–1.03); fatal stroke: HR: 0.71 (0.39–1.30); HF hospitalisation: HR: 0.94 (0.78–1.13); ACS hospitalisation: HR: 1.05 (0.94–1.18)	CV death: HR: 0.49 (0.27–0.92); non-fatal MI: HR: 1.18 (0.73–1.90); non‑fatal stroke: HR: 0.74 (0.35–1.57); all‑cause death: HR: 0.51 (0.31–0.84); HF hospitalisation: HR: 0.86 (0.48–1.55); unstable‑angina hospitalisation: HR: 1.56 (0.60–4.01)	CV death: HR: 0.78 (0.66–0.93), *P* = 0.007; non-fatal MI: HR: 0.86 (0.73–1.00), *P* = 0.046; non‑fatal stroke: HR: 0.89 (0.72–1.11), *P* = 0.30; all‑cause death: HR: 0.85 (0.74–0.97), *P* = 0.02; HF hospitalisation: HR: 0.87 (0.73–1.05), *P* = 0.14	CV death: HR: 0.98 (0.65–1.48); non-fatal MI: HR: 0.74 (0.51–1.08); non‑fatal stroke: HR: 0.61 (0.38–0.99); all‑cause death: HR: 1.05 (0.74–1.50); HF hospitalisation: HR: 1.11 (0.77–1.61)	CV death: HR: 0.88 (0.72–1.08); non-fatal MI: HR: 0.85 (0.71–1.01); non‑fatal stroke: HR: 0.82 (0.66–1.01); all‑cause death: HR: 0.83 (0.71–0.98); HF hospitalisation: HR: 0.93 (0.77–1.12)	CV death: HR: 0.94 (0.78–1.13); MI: HR: 1.03 (0.87–1.22); stroke: HR: 1.12 (0.79–1.58); all-cause death: HR: 0.94 (0.78–1.13); HF hospitalisation: HR: 0.96 (0.75–1.23)
Serious adverse events	GI events in 47.4 vs. 34.1% (*P* < 0.0001); no differences in pancreatitis, cancer, severe hypoglycaemia or other serious events	Confirmed pancreatitis 0.4% vs. 0.3%, pancreatic cancer 0.3% vs. 0.2%, severe hypoglycaemia 7.9% vs. 7.7%	GI events more frequent with semaglutide; no significant differences in pancreatitis, pancreatic cancer or severe hypoglycaemia	GI events more frequent with liraglutide; no significant differences in pancreatitis, cancer or severe hypoglycaemia	GI events more frequent with semaglutide; increased diabetic retinopathy (HR: 1.76, 95% CI: 1.11–2.78); no differences in pancreatitis or cancer	Nausea and vomiting more frequent with semaglutide; no significant increase in pancreatitis, cancer or severe hypoglycaemia	GI events more frequent with lixisenatide; no increase in pancreatitis, cancer or severe hypoglycaemia
RoB 2 risk of bias for MACE	Low risk	Low risk	Low risk	Low risk	Low risk	Low risk	Low risk
Funding source	Eli Lilly and Company	AstraZeneca	Novo Nordisk	Novo Nordisk	Novo Nordisk	Novo Nordisk	Sanofi

ACE, angiotensin-converting enzyme; ARBs, angiotensin II receptor blockers; CV, cardiovascular; GI, gastrointestinal; HF, heart failure; MACE, major adverse cardiovascular events; MI, myocardial infarction; RCT, randomised controlled trial; SC, subcutaneous; SGLT2, sodium-glucose co-transporter 2; T2DM, type 2 diabetes.

We included seven RCTs [7,11–15] that evaluated the cardiovascular efficacy of GLP-1RAs in adults with T2DM and ASCVD. All were large, multicentre phase 3 or 4 studies conducted across diverse geographical regions, with sample sizes ranging from just over 3000 to nearly 15 000 participants.

The interventions assessed were liraglutide, injectable semaglutide, dulaglutide, exenatide, lixisenatide and oral semaglutide, each compared with placebo on top of standard care. Median follow-up varied between 1.3 and 5.4 years.

All trials enrolled adults with T2DM and a history of ASCVD or multiple cardiovascular risk factors. Most participants were receiving background cardioprotective therapy, such as statins, angiotensin-converting enzyme inhibitors and antiplatelet agents. Women accounted for roughly 30–46% of study cohorts, and the mean age ranged from 61 to 66 years.

### Primary composite outcome of major adverse cardiovascular events

**Fig. 2:**
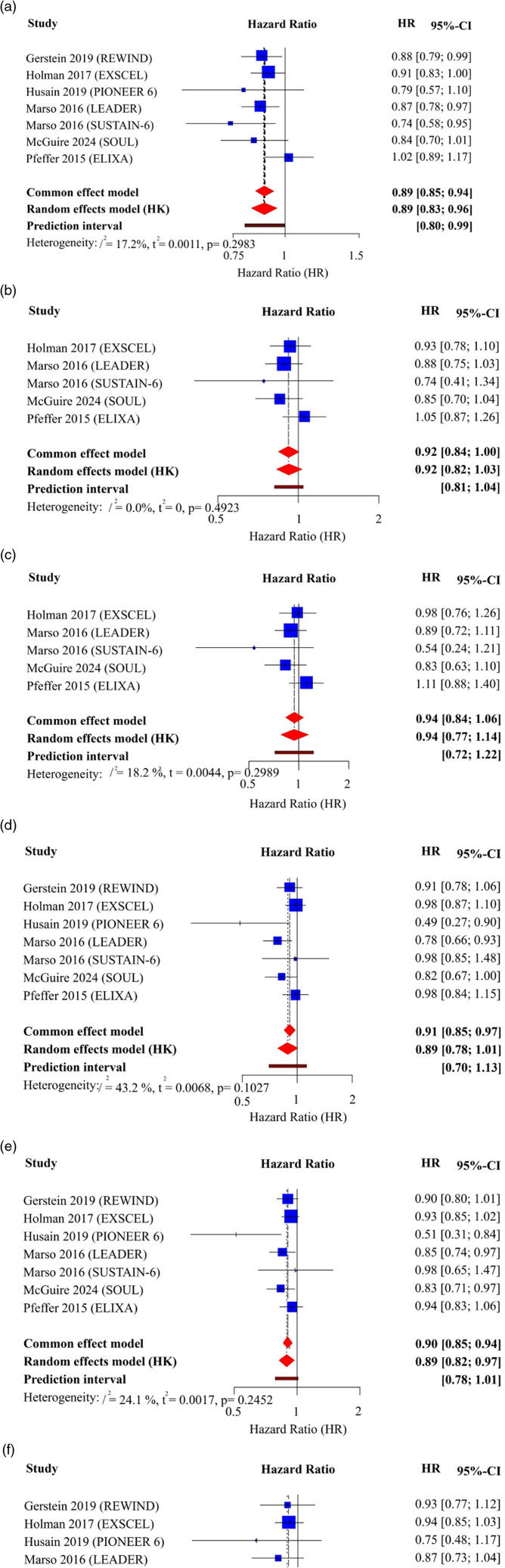
Forest plots comparing GLP-1 receptor agonists with placebo or usual care for key cardiovascular outcomes. (a) Primary composite of MACE. (b) Fatal MI. (c) Fatal stroke. (d) Cardiovascular mortality. (e) All‑cause mortality. (f) Hospitalisation for heart failure. Squares represent individual trial estimates (size proportional to inverse variance); horizontal lines depict 95% CIs; diamonds indicate pooled HRs. A HR < 1 favours GLP‑1 receptor agonists. ASCVD, atherosclerotic cardiovascular disease; CI, confidence interval; GLP‑1RA, glucagon‑like peptide‑1 receptor agonist; HF, heart failure; HR, hazard ratio; MACE, major adverse cardiovascular events; MI, myocardial infarction.

Seven RCTs were included, comprising a total of 56 191 participants. The random-effects model yielded a pooled hazard ratio of 0.89 (95% CI: 0.83–0.96), indicating an 11% relative risk reduction in favour of GLP-1RAs compared to placebo. Heterogeneity was low (*I*^2^ = 17.2%; *τ*^2^ = 0.0011; *P* = 0.30, Fig. 2a). The overall certainty of the evidence was high.

### Acute myocardial infarction

Five RCTs contributed data from 43 107 participants. The random-effects model showed a pooled hazard ratio of 0.92 (95% CI: 0.82–1.03), suggesting an 8% relative reduction with GLP-1RAs compared to placebo. However, the CI includes the null value, and thus the effect is not statistically significant. Heterogeneity was absent (*I*^2^ = 0%; *τ*^2^ = 0; *P* = 0.49; Fig. 2b), indicating no meaningful variability across studies. The overall certainty of the evidence was moderate.

The analysis for nonfatal MI is presented in Supplementary Fig. S1, Supplemental digital content 1, https://links.lww.com/CAEN/A80. This included six studies with 53 008 participants and yielded a hazard ratio of 0.91 (95% CI: 0.83–1.00), with low heterogeneity (*I*^2^ = 14.1%). The overall certainty of the evidence was moderate.

### Stroke

Five RCTs provided data from 43 107 participants. The random-effects model yielded a pooled hazard ratio of 0.94 (95% CI: 0.77–1.14; Fig. 2c), suggesting a 6% relative reduction with GLP-1RAs versus placebo; however, the CI includes the null value, rendering the effect statistically nonsignificant. Heterogeneity was low (*I*^2^ = 18.2%; *τ*^2^ = 0.0044; *P* = 0.30), indicating minimal variability between studies. The overall certainty of the evidence was moderate.

The analysis of nonfatal stroke is presented in Supplementary Fig. S2, Supplemental digital content 1, https://links.lww.com/CAEN/A80. This included seven studies with 56 191 participants and produced a hazard ratio of 0.89 (95% CI: 0.75–1.06), with moderate heterogeneity (*I*^2^ = 44.4%). The overall certainty of the evidence was low.

### Cardiovascular mortality

Seven RCTs were included, encompassing a total of 56 191 participants. The random-effects model estimated a pooled hazard ratio of 0.89 (95% CI: 0.78–1.01; Fig. 2d), suggesting an 11% relative reduction in favour of GLP-1RAs compared to placebo; however, the CI includes the null value, and the effect does not reach statistical significance. Heterogeneity was moderate (*I*^2^ = 43%; *τ*^2^ = 0.0068; *P* = 0.10), indicating some variability across studies. The overall certainty of the evidence was low.

### All-cause mortality

Seven RCTs contributed data from 56 191 participants. The random-effects model estimated a pooled hazard ratio of 0.89 (95% CI: 0.82–0.97; Fig. 2e), reflecting an 11% relative risk reduction in favour of GLP-1RAs over placebo. Heterogeneity was low (*I*^2^ = 24.1%; *τ*^2^ = 0.0017; *P* = 0.25), indicating limited variability between studies. The overall certainty of the evidence was high.

### Hospitalisation for heart failure

Seven RCTs were analysed, comprising 56 191 participants. The random-effects model yielded a pooled hazard ratio of 0.93 (95% CI: 0.89–0.98; Fig. 2f), indicating a 7% relative reduction in favour of GLP-1RAs compared to placebo. Heterogeneity was null (*I*^2^ = 0%; *τ*^2^ = 0; *P* = 0.90), supporting the consistency of effect across studies. The overall certainty of the evidence was high.

### Subgroup analysis

The therapeutic effect differed slightly between human-sequence GLP-1RAs (liraglutide, semaglutide and dulaglutide) and exendin-4–based agents (exenatide and lixisenatide). The five trials evaluating human-based compounds demonstrated a relative risk reduction of 14% (hazard ratio = 0.86; 95% CI: 0.80–0.92), with no appreciable heterogeneity (*I*^2^ = 0%). In contrast, the two studies assessing exendin-4 derivatives yielded a pooled estimate close to the null (hazard ratio = 0.94; 95% CI: 0.87–1.02) and moderate heterogeneity (*I*^2^ = 45%). The test for interaction between subgroups was not statistically significant (*χ*^2^ = 3.05; *P* = 0.08), suggesting that the observed difference in effect size may be because of chance (Supplementary Fig. S3, Supplemental digital content 1, https://links.lww.com/CAEN/A80).

The three studies with greater than or equal to 3 years of follow-up (24 891 participants) showed a 13% relative risk reduction (hazard ratio = 0.87; 95% CI: 0.83–0.91) with no heterogeneity (*I*^2^ = 0%). The four trials with less than 3 years of follow-up (31 300 participants) demonstrated a slightly attenuated yet protective effect (hazard ratio = 0.92; 95% CI: 0.86–0.99), with moderate heterogeneity (*I*^2^ = 44%). The interaction test between subgroups was NS (*χ*^2^ = 0.40; *P* = 0.53), indicating that the duration of follow-up did not conclusively modify the magnitude of the observed benefit of GLP-1RAs (Supplementary Fig. S4, Supplemental digital content 1, https://links.lww.com/CAEN/A80).

### Risk of bias

**Fig. 3:**
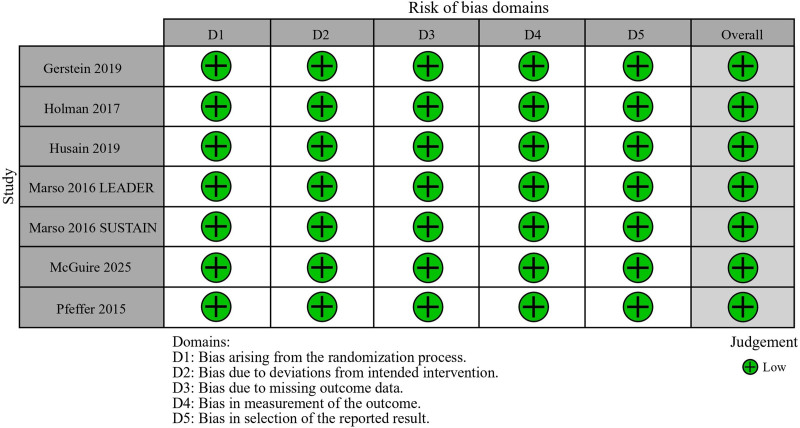
Risk-of-bias assessment using the RoB 2 tool for the outcome MACE. Traffic‑light plot summarising judgements across five domains – randomisation (D1), deviations from intended interventions (D2), missing outcome data (D3), outcome measurement (D4), and selection of the reported result (D5) – for each included trial. Green = low risk; yellow = some concerns; red = high risk; all studies were rated overall as low risk of bias. MACE, major adverse cardiovascular events.

All included trials were assessed using the RoB 2 tool and were judged to be at low risk of bias across all five domains: randomisation process (D1), deviations from intended interventions (D2), missing outcome data (D3), measurement of the outcome (D4) and selection of the reported result (D5), as well as for the overall risk of bias (Fig. 3).

### Publication bias

The funnel plot (Supplementary Fig. S5, Supplemental digital content 1, https://links.lww.com/CAEN/A80) showed a visually symmetrical distribution of the seven trials, with no apparent clustering of small studies with extreme effects to suggest publication bias. As the number of studies was fewer than 10 (*k* = 7), formal statistical tests for small-study effects were not performed because of limited power. Consequently, the assessment was restricted to a qualitative inspection, which indicated a low likelihood of bias.

## Discussion

This systematic review and meta-analysis included seven RCTs comprising 56 191 adults T2DM and ASCVD. Our findings suggest that some GLP-1RAs confer a clinically relevant cardiovascular benefit compared with placebo. GLP-1RAs were associated with an 11% relative reduction in MACE (hazard ratio: 0.89; 95% CI 0.83–0.96; *I*^2^ = 17%), accompanied by reductions of similar magnitude in all-cause mortality and a 7% decrease in the risk of hospitalisation for heart failure. Point estimates for fatal MI, fatal stroke and cardiovascular mortality also favoured GLP-1RAs, although the CIs crossed unity owing to the smaller absolute number of events. According to GRADE, the certainty of evidence was high for MACE, all-cause mortality and heart-failure hospitalisation, and ranged from moderate to low for the remaining outcomes because of imprecision or moderate heterogeneity.

In our subgroup analysis, cardiovascular benefit appeared more consistent among long-acting human-sequence GLP-1RAs, whereas exendin-based agents showed estimates closer to neutrality. The inclusion of SOUL further clarifies that oral administration does not preclude cardiovascular benefit, addressing an important unmet need for patients with T2D who are reluctant to initiate injectable therapy and may prefer an oral alternative with proven macrovascular efficacy. Therefore, the incremental value of this review lies in positioning SOUL as more than an additional trial: it allows the GLP-1RA evidence base to be reinterpreted according to molecular backbone and route of administration, rather than as a single homogeneous drug class [7].

When translated into absolute terms, the magnitude of benefit varied according to baseline risk, follow-up duration, and the specific GLP-1RA evaluated. Across individual trials, the absolute risk reduction for MACE ranged from no apparent benefit with lixisenatide in ELIXA to 2.3% with injectable semaglutide in SUSTAIN-6. The corresponding trial-specific number needed to treats were approximately 44 for SUSTAIN-6 over 2.1 years, 53 for LEADER over 3.8 years, 56 for SOUL over 49.5 months, 72 for REWIND over 5.4 years and 125 for EXSCEL over 3.2 years. These findings indicate that, although the pooled relative effect was modest, the absolute clinical benefit may be meaningful in high-risk populations, particularly when baseline cardiovascular risk is elevated and treatment is sustained over several years [16–19].

The magnitude of risk reduction for MACE aligns with recent systematic reviews, which report pooled relative risks between 0.88 and 0.92 [16,17]. Meta-analyses focusing solely on semaglutide show more pronounced benefits [18], consistent with our subgroup analysis: the five trials evaluating human-sequence analogues (liraglutide, semaglutide and dulaglutide) demonstrated a greater risk reduction than the two trials of exendin-4 derivatives (exenatide and lixisenatide). For non-fatal stroke, the 11% reduction observed (hazard ratio: 0.89) mirrors the 14% decrease reported by Stefanou *et al*. [18].

Although statistical heterogeneity for the primary MACE outcome was low, this should not be interpreted as evidence that the included trials were clinically homogeneous. Important differences existed in baseline cardiovascular risk, acuity of disease, inclusion criteria, follow-up duration, background cardioprotective therapy and GLP-1RA molecule evaluated. ELIXA enrolled patients shortly after an acute coronary syndrome, representing an unstable, postevent population in whom lixisenatide showed a neutral effect. In contrast, REWIND included a broader and comparatively lower-risk population, with only approximately one third of participants having established cardiovascular disease, yet showed benefit over the longest follow-up period. SOUL, meanwhile, enrolled a contemporary high-risk population with established ASCVD, chronic kidney disease, or both, with greater use of background cardioprotective therapies, including sodium-glucose co-transporter 2 inhibitors. These differences suggest that the observed treatment effect should be interpreted in light of clinical context rather than relying solely on *I*^2^ statistics. Low statistical heterogeneity may therefore mask clinically meaningful variation across trials, particularly regarding patient risk profile, timing after cardiovascular events, competing background therapies, molecular structure and route of administration.

The therapeutic effect appeared numerically more favourable among human-sequence GLP-1RAs than among exendin-4-based agents. The five trials evaluating human-sequence compounds showed a pooled hazard ratio of 0.86 (95% CI: 0.80–0.92), whereas the two trials evaluating exendin-4 derivatives yielded a pooled hazard ratio of 0.94 (95% CI: 0.87–1.02). However, the test for interaction did not reach statistical significance (*χ*^2^ = 3.05; *P* = 0.08). Therefore, this subgroup finding should be interpreted cautiously as hypothesis-generating rather than conclusive evidence of differential cardiovascular efficacy by molecular backbone.

Although the point estimates suggested a more consistent MACE reduction among human-sequence GLP-1RAs compared with exendin-4-based agents, this apparent difference was not supported by a statistically significant interaction test. Therefore, the observed pattern should not be interpreted as definitive evidence that molecular backbone modifies cardiovascular efficacy. The finding may reflect true pharmacologic differences, but it may also be influenced by differences in trial populations, follow-up duration, background therapy, event rates, or limited statistical power, particularly because only two exendin-based trials were available. Accordingly, this subgroup analysis should be viewed as exploratory and hypothesis-generating, warranting further evaluation in future individual-patient-data meta-analyses or adequately powered comparative studies.

Mechanistically, oral or subcutaneous GLP-1RAs promote weight loss, lower blood pressure, improve lipid profiles, attenuate systemic inflammation and exert direct anti-atherogenic effects on the endothelium and myocardium. These actions plausibly underlie the reduction in atherothrombotic events, whereas the modest but significant reduction in heart-failure hospitalisation may reflect haemodynamic and natriuretic effects rather than reverse cardiac remodelling [6].

## Strengths and limitations

Key strengths of this review include the inclusion of large, low-risk RCTs; minimal heterogeneity for the primary outcome; prespecified subgroup analyses; and a rigorous GRADE assessment. Limitations comprise the low absolute number of fatal events, which reduced precision for fatal MI, fatal stroke and cardiovascular mortality; the aggregation of agents with heterogeneous pharmacokinetics; and relatively short follow-up (<4 years in most trials), potentially under- or overestimating benefits apparent only with longer exposure or head-to-head comparisons. A further limitation is that low statistical heterogeneity may underestimate clinically relevant variation across trials, since populations differed substantially in baseline cardiovascular risk, timing of prior cardiovascular events, the prevalence of established ASCVD or CKD, background cardioprotective therapy, follow-up duration and GLP-1RA structure.

## Implications for practice and research

Future research should focus on long-term cardiovascular outcome trials, stratifying by clinically relevant covariates – such as prior atherosclerotic events, stage of chronic kidney disease and left ventricular function – and quantifying dose–response relationships and interactions between GLP-1RAs and background therapies. Such work will refine absolute risk estimates for infrequent yet critical outcomes.

### Conclusion

In adults with T2DM and established ASCVD, oral or subcutaneous GLP-1RAs appear to significantly reduce MACE, all-cause mortality and heart-failure hospitalisation, with favourable – albeit less precise – effects on fatal MI, fatal stroke and cardiovascular mortality. These biologically plausible findings, consistent with prior literature, support the preferential use of GLP-1RAs in this high-risk population.

## Acknowledgements

Guarantor for the paper is G.C.G.-G.

Additional information is available from the corresponding author upon reasonable request. The review protocol is publicly registered in PROSPERO (CRD420251034652; https://www.crd.york.ac.uk/PROSPERO).

### Conflicts of interest

There are no conflicts of interest.

## Supplementary Material
