# Supplementary material for: Effect of glucagon-like peptide-1 receptor agonists on major adverse cardiovascular events in adults with type 2 diabetes and established atherosclerotic cardiovascular disease: systematic review and meta-analysis of randomised trials
Source: Cardiovasc Endocrinol Metab. 2026 Jun 26;15(3):e00360. doi: 10.1097/XCE.0000000000000360 (PMC13313748; doi:10.1097/XCE.0000000000000360)
Supplement: Supplementary file 1 [file xce-15-e00360-s001.docx]

**Supplementary Material**

**Search logs**

**1. PubMed**

| **Characteristic** | **Report** |
| --- | --- |
| Search type | New |
| Database | Medline |
| Platform | PubMed |
| Data searched | 06 May 2025 |
| Data range | No restriction -06 May 2025 |
| Language limits | English and Spanish |
| Other limits | None |
| Search strategy | \| **Element** \| **Search terms** \| \| --- \| --- \| \| **Population** \| (“Diabetes Mellitus, Type 2”[MeSH] OR “type 2 diabetes” OR T2DM) AND (“Cardiovascular Diseases”[MeSH] OR “Atherosclerosis”[MeSH] OR “atherosclerotic cardiovascular disease” OR “cardiovascular event*”) \| \| **Intervention** \| “Glucagon‑Like Peptide 1 Receptor Agonists”[MeSH] OR “Glucagon‑Like Peptide 1”[MeSH] OR “GLP‑1 receptor agonist” OR liraglutide OR semaglutide OR dulaglutide OR exenatide OR lixisenatide \| \| **Outcomes** \| “Cardiovascular Diseases/mortality”[MeSH] OR “Stroke”[MeSH] OR “Myocardial Infarction”[MeSH] OR “Heart Failure”[MeSH] OR “Hospitalization”[MeSH] OR “Mortality”[MeSH] OR MACE OR “major adverse cardiovascular events” OR “cardiovascular mortality” OR “all‑cause mortality” \| \| **Study type** \| “Randomised Controlled Trial”[Publication Type] OR randomised OR randomized OR RCT OR CVOT \| |
| Number of records identified: | 337 (duplicates removed: 337) |

1. **Embase/Clinical Trials**

| **Characteristic** | **Report** |
| --- | --- |
| Search type | New |
| Database | Embase, Clinical Trials |
| Platform | Elsevier |
| Data searched | 06 May 2025 |
| Search range | No restriction - 06 May 2025 |
| Language limits | English and Spanish |
| Other limits | Article, Review, Clinical Trial |
| Search strategy | \| **Element** \| **Search terms** \| \| --- \| --- \| \| **Population** \| ('type 2 diabetes mellitus'/exp OR 'type 2 diabetes' OR T2DM) AND ('cardiovascular disease'/exp OR 'atherosclerosis'/exp OR 'atherosclerotic cardiovascular disease' OR 'cardiovascular event*') \| \| **Intervention** \| 'glucagon like peptide 1 receptor agonist'/exp OR 'glucagon like peptide 1'/exp OR 'GLP-1 receptor agonist' OR liraglutide OR semaglutide OR dulaglutide OR exenatide OR lixisenatide \| \| **Outcomes** \| 'major cardiovascular event' OR 'cardiovascular mortality'/exp OR 'stroke'/exp OR 'myocardial infarction'/exp OR 'heart failure'/exp OR 'hospitalization'/exp OR 'mortality'/exp OR MACE OR "all-cause mortality" \| \| **Study type** \| 'randomized controlled trial'/exp OR 'clinical trial'/exp OR randomized:ti,ab OR randomised:ti,ab OR rct:ti,ab OR cvot:ti,ab \| \| **Sources** \| ([embase]/lim OR 'clinical trial':dtype) AND ('article'/it OR 'clinical trial'/it OR 'review'/it) \| |
| Number of records identified: | 1253, (duplicates removed: 682) |

1. **Scopus**

| **Characteristic** | **Report** |
| --- | --- |
| Search type | New |
| Database | Scopus |
| Platform | Scopus |
| Data searched | 06 May 2025 |
| Data range | No restriction - 06 May 2025 |
| Language limits | English and Spanish |
| Other limits | Journal, Article, Review |
| Search strategy | \| **Element** \| **Search terms** \| \| --- \| --- \| \| **Population** \| ("type 2 diabetes" OR T2DM) AND ("cardiovascular disease" OR "atherosclerotic cardiovascular disease" OR "cardiovascular event" OR atherosclerosis) \| \| **Intervention** \| ("GLP-1 receptor agonist" OR "glucagon like peptide 1 receptor agonist" OR liraglutide OR semaglutide OR dulaglutide OR exenatide OR lixisenatide) \| \| **Outcomes** \| ("major adverse cardiovascular events" OR MACE OR "cardiovascular mortality" OR "myocardial infarction" OR "stroke" OR "heart failure" OR "hospitalization" OR "all-cause mortality" OR mortality) \| \| **Study type** \| ("randomized controlled trial" OR "randomised controlled trial" OR rct OR cvot OR randomized OR randomised OR "clinical trial") \| |
| Number of records identified | 987, (duplicates removed: 795 |

1. **Cochrane CENTRAL**

| **Characteristic** | **Report** |
| --- | --- |
| Search type | New |
| Database | Cochrane Central Register of Controlled Trials |
| Platform | Ovid |
| Data searched | 06 May 2025 |
| Data range | No restriction - 06 May 2025 |
| Language limits | English and spanish |
| Other limits | journal article |
| Search strategy | \| **Element** \| **Search terms** \| \| --- \| --- \| \| **Population** \| (type 2 diabetes OR T2DM).mp.  AND  (cardiovascular disease OR atherosclerosis OR "atherosclerotic cardiovascular disease" OR "cardiovascular event*").mp. \| \| **Intervention** \| ("glucagon-like peptide 1 receptor agonist" OR GLP-1 OR liraglutide OR semaglutide OR dulaglutide OR exenatide OR lixisenatide).mp. \| \| **Outcomes** \| ("major adverse cardiovascular events" OR MACE OR "myocardial infarction" OR stroke OR "heart failure" OR hospitalization OR "all-cause mortality" OR "cardiovascular mortality").mp. \| |
| Number of records idenfified | 221, (duplicates removed: 213 |

**Figure S1. Effect of GLP‑1 receptor agonists on non‑fatal myocardial infarction**


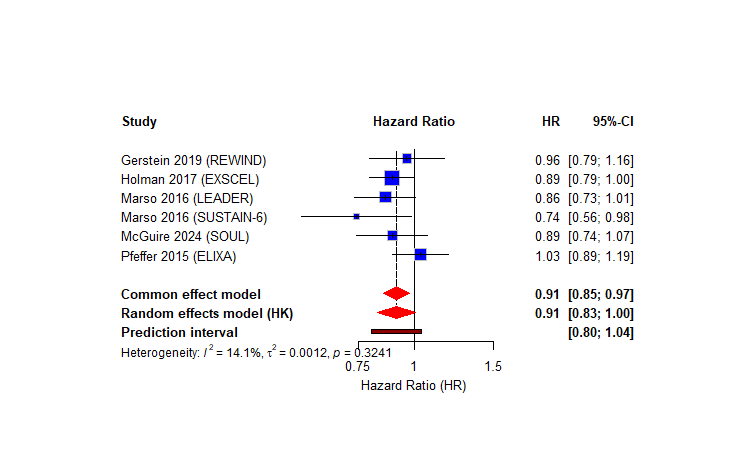


**Figure S2. Effect of GLP‑1 receptor agonists on non‑fatal strok**
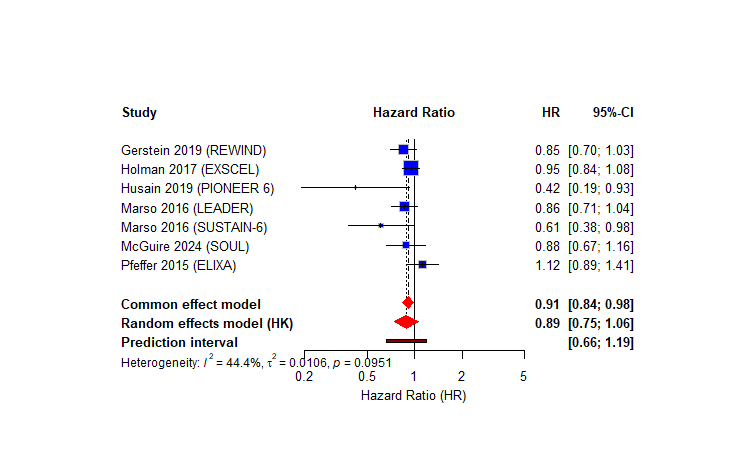


**Figure S3. Subgroup analysis by GLP‑1 receptor agonist class: human‑sequence analogues vs exendin‑4 derivatives**


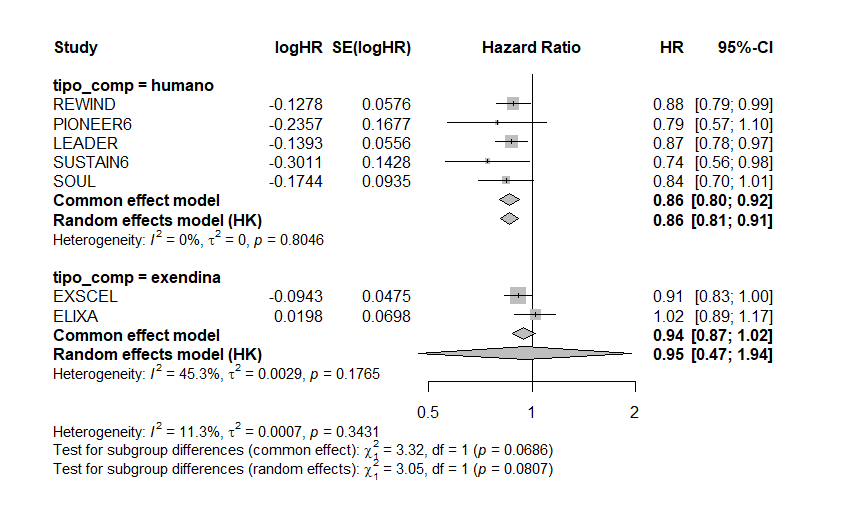


**Figure S4. Subgroup analysis by follow‑up duration: < 3 years vs ≥ 3 years**


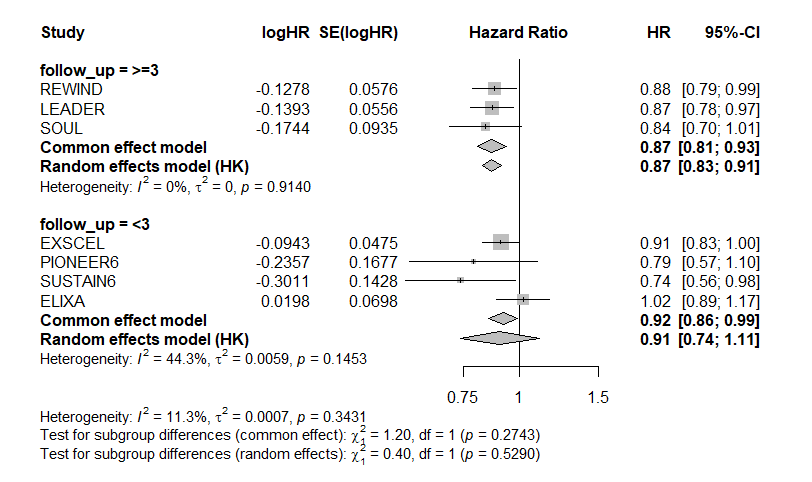


**Figure S5. Funnel plot for qualitative assessment of publication bias**


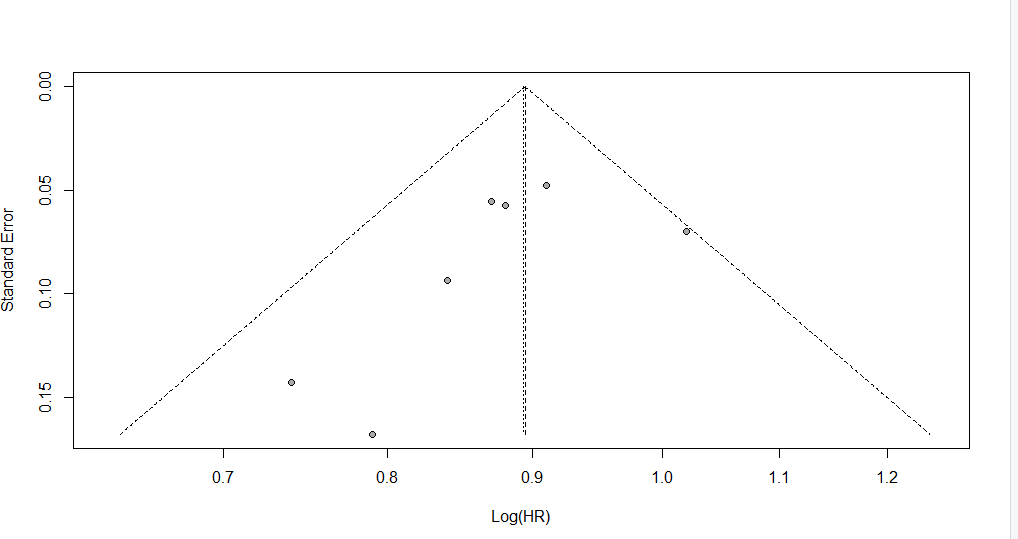


**Overall certainty of evidence (GRADE)**

**Question:** GLP‑1 receptor agonists versus placebo or standard care without GLP‑1RAs in adults with type 2 diabetes and established atherosclerotic cardiovascular disease (ASCVD)

| **Certainty assessment** | | | | | | | **No. of patients** | | **Effect** | | **Certainity** | **Importance** |
| --- | --- | --- | --- | --- | --- | --- | --- | --- | --- | --- | --- | --- |
| **No. of studies** | **Study design** | **Risk of bias** | **Inconsistency** | **Indirectness** | **Imprecision** | **Other considerations** | **GLP‑1 receptor agonists** | **Placebo or standard care without GLP‑1RA** | **Relative (95 % CI)** | **Absolute (95 % CI)** |  |  |
| **MACE** | | | | | | | | | | | | |
| 7 | RCTs | Not serious | Not serious | Not serious | Not serious | None | 3195/28071 (11.4%) | 3551/28120 (12.6%) | **HR 0.89** (0.83 a 0.96) | **13 fewer per 1 000 (from 20 fewer to 5 fewer)** | ⨁⨁⨁⨁ High | CRITICAL |
| **Fatal myocardial infarction** | | | | | | | | | | | | |
| 5 | RCTs | Not serious | Not serious | Not serious | serious^a^ | None | 492/21553 (2.3%) | 536/21554 (2.5%) | **HR 0.92** (0.82 a 1.03) | **2 fewer per 1 000** (from 4 fewer to 1 more) | ⨁⨁⨁◯ Moderated^a^ | CRITICAL |
| **Non‑fatal myocardial infarction** | | | | | | | | | | | | |
| 6 | RCTs | Not serious | Not serious | Not serious | serious^a^ | None | 1244/25037 (5.0%) | 1370/25086 (5.5%) | **HR 0.91** (0.83 a 1.00) | **5 fewer per 1 000**  (from 9 fewer to 0 fewer) | ⨁⨁⨁◯ Moderated^a^ | CRITICAL |
| **Fatal stroke** | | | | | | | | | | | | |
| 5 | RCTs | Not serious | Not serious | Not serious | serious ^a^ | None | 162/21553 (0.8%) | 172/21554 (0.8%) | **HR 0.94** (0.77 a 1.14) | **0 fewer per 1 000**  (from 2 fewer to 1 more) | ⨁⨁⨁◯ Moderated^a^ | CRITICAL |
| **Non-fatal stroke** | | | | | | | | | | | | |
| 7 | RCTs | Not serious | serious^b^ | Not serious | serious^a^ | None | 702/28071 (2.5%) | 831/28120 (3.0%) | **HR 0.89** (0.75 a 1.06) | **3 fewer per 1,000** (from 7 fewer to 2 more) | ⨁⨁◯◯ Low^,b^ | CRITICAL |
| **Cardiovascular mortality** | | | | | | | | | | | | |
| 7 | RCTs | Not serious | serious^b^ | Not serioue | serious^a^ | None | 1392/28071 (5.0%) | 15520/28120 (55.2%) | **HR 0.89** (0.78 a 1.01) | **41 fewer per 1,000** (from 87 fewer to 4 more) | ⨁⨁◯◯ Low^,b^ | CRITICAL |
| **All-cause mortality** | | | | | | | | | | | | |
| 7 | RCTs | Not serious | Not serious | Not serious | Not serious | None | 2258/28071 (8.0%) | 2528/28120 (9.0%) | **HR 0.89** (0.82 a 0.97) | **9 fewer per 1,000** (from 16 fewer to 3 fewer) | ⨁⨁⨁⨁ High | CRITICAL |
| **Hearth failure hospitalisation** | | | | | | | | | | | | |
| 7 | RCTs | Not serious | Not serious | Not serious | Not serious | None | 938/28071 (3.3%) | 1077/28120 (3.8%) | **HR 0.93** (0.89 a 0.98) | **3 fewer per 1,000** (from 4 fewer to 1 fewer) | ⨁⨁⨁⨁ High | CRITICAL |

**CI**: Confidence interval  **HR**: Hazard ratio

Explanations

a. CI crosses the line of no effect

b. I² = 44 %
